# *CHSY3* can be a Poor Prognostic Biomarker and Mediates Immune Evasion in Stomach Adenocarcinoma

**DOI:** 10.3389/fgene.2022.876588

**Published:** 2022-04-27

**Authors:** Xinwei Li, Yongfei Fan, Yue Zhang, Yanyan Wang, Menglin Zhao, Mingyue Tang, Huiyuan Li, Jiaqi Mi, Zhijun Geng, Zishu Wang, Fang Su

**Affiliations:** ^1^ Department of Medical Oncology, First Affiliated Hospital of Bengbu Medical College, Bengbu, China; ^2^ Department of Thoracic Surgery, The Affiliated Changzhou No. 2 People’s Hospital of Nanjing Medical University, Changzhou, China; ^3^ Department of Central Laboratory, First Affiliated Hospital of Bengbu Medical College, Bengbu, China

**Keywords:** *CHSY3*, biomarker, immune evasion, prognosis, immunotherapy, stomach adenocarcinoma

## Abstract

**Background:** Chondroitin sulphate synthase 3 (*CHSY3*) is an important enzyme that regulates glycosylation, but it has not been reported in tumours. This study explored for the first time the oncological features of *CHSY3* in stomach adenocarcinoma (STAD).

**Methods:** We analysed *CHSY3* expression in STAD through the Cancer Genome Atlas (TCGA) database and verified our findings by immunohistochemical staining and Western blot experiments. The prognostic value of *CHSY3* in STAD was analysed through the biological aspects of *CHSY3* in STAD, such as communal clinical follow-up survival data, methylation sites, tumour immune microenvironment (TIME) and immune cell surface checkpoints. Finally, the immune-evasion potential of *CHSY3* in STAD was assessed on the Tumor Immune Dysfunction and Exclusion (TIDE) website and immunohistochemical staining experiment.

**Results:** *CHSY3* overexpression in STAD was associated with a poor prognosis based on immunohistochemical staining and Western blot experiments. Multivariate Cox analysis suggested that *CHSY3* could be an independent prognostic risk factor. Pathway enrichment and TIME analysis demonstrated that *CHSY3* up-regulated mesenchymal activation and immune activation signals in STAD, while TIDE assessment revealed that the risk of immune evasion was significantly higher in the high *CHSY3* expression group than in the low *CHSY3* expression group. Risk model scores based on *CHSY3*-associated immune cell surface checkpoints also presented poor prognosis, and immune evasion was significantly higher in the high-risk group than in the low-risk group.

**Conclusions:** This study analysed *CHSY3* from multiple biological perspectives and revealed that *CHSY3* can be a biomarker of poor prognosis and mediates the TIME immune-evasion status in STAD.

## Introduction

Stomach cancer is the fifth most common cancer worldwide and has the third-highest mortality rate of all cancers (Smyth et al., 2020). More than one million cases of stomach cancer are diagnosed worldwide annually, and approximately 780,000 people die from it (Bray et al., 2018). Currently, gastric cancer is mainly diagnosed histopathologically after endoscopic biopsy, and clinical staging is determined by computed tomography, positron emission tomography-computed tomography and other methods (Ajani et al., 2016). Since the early symptoms of gastric cancer are not noticeable, most cases are already in advanced stages at the time of diagnosis; thus, the prognosis of gastric cancer is still very poor (Thrift and El-Serag, 2020). At present, molecular targeted therapy, tumour immunotherapy and gene therapy are continuously applied in the diagnosis and treatment of gastric cancer and showed promising results (Joshi and Badgwell, 2021). Therefore, further search for highly specific molecular biomarkers or targets for drug therapy is important.

Chondroitin sulphate synthase 3 (*CHSY3*) is a glycosyltransferase with glucuronosyltransferase and N-acetylgalactosaminyltransferase activities (Yada et al., 2003). Glycosylation of proteins is one of the most common post-translational modifications of proteins (Schjoldager et al., 2020). It is the process of transferring sugars to proteins and specific amino acid residues on proteins to form glycosidic bonds under the action of glycosyltransferases (Schjoldager et al., 2020). Cell carcinogenesis is often accompanied by structural changes in the glycoconjugate chain, such as the appearance of repetitive N-acetylamino galactose structures at the branch ends of the glycoconjugate chain and an increase in the levels of sialic acid and fucose (Stowell et al., 2015; Oliveira-Ferrer et al., 2017), which are commonly used as tumour markers in clinical practice (Wang et al., 2019; Luo et al., 2021). Glycosyltransferases are indirectly related to the development of malignant tumours and the prognosis of patients with cancer by altering the sugar chain (Silsirivanit, 2019). In addition, abnormal alterations in tumour cell surface glycosylation also lead to tumour immune microenvironment (TIME) immune evasion, thus providing a new immune checkpoint (IC) for immunotherapy (RodrÍguez et al., 2018; Bartish et al., 2020).

According to the pathological staging of gastric cancer, stomach adenocarcinoma (STAD) accounts for 95% of all gastric cancer cases (Ferlay et al., 2010). In this study, we explored the prognostic value of *CHSY3* in STAD and determined whether it can be used as a biomarker of STAD prognosis. We also analysed the effect of *CHSY3* on the TIME of STAD and explored its implications for STAD immunotherapy.

## Materials and Methods

### Acquisition of *CHSY3* Expression Profiles

We selected the fragments per kilobase of transcript per million mapped read format of pan-cancer from The Cancer Genome Atlas (TCGA) database (https://www.cancer.gov/) (tumour, *n* = 10363; normal, *n* = 730) using the Sento Academic website (https://www.xiantao.love/) to analyse the differential expression of *CHSY3* in pan-cancer (*p*-value < 0.05; Wilcoxon rank-sum test). Then, STAD expression matrix files (tumour, *n* = 375; normal, *n* = 32) were downloaded from TCGA database for paired and unpaired difference analyses by using the “limma” R package (*p*-value < 0.05; t-test). Table 1 exhibits the clinical information of high and low *CHSY3* expression groups in STAD in TGCA database.

**TABLE 1:** Clinical information of CHSY3 high and low expression groups in the TCGA database.

Characteristic (n)	Low expression	High expression
	*n* = 187	*n* = 188
T stage, n (%)		
T1	16 (4.4%)	3 (0.8%)
T2	45 (12.3%)	35 (9.5%)
T3	88 (24%)	80 (21.8%)
T4	38 (10.4%)	62 (16.9%)
unkown	0 (0%)	8 (2.1%)
N stage, n (%)		
N0	58 (16.2%)	53 (14.8%)
N1	47 (13.2%)	50 (14%)
N2	39 (10.9%)	36 (10.1%)
N3	37 (10.4%)	37 (10.4%)
Unkown	6 (1.6%)	12 (3.2%)
M stage, n (%)		
M0	165 (46.5%)	165 (46.5%)
M1	14 (3.9%)	11 (3.1%)
Unkown	8 (2.1%)	11 (2.9%)
Stage, n (%)		
Stage I	34 (9.7%)	19 (5.4%)
Stage II	52 (14.8%)	59 (16.8%)
Stage III	74 (21%)	76 (21.6%)
Stage IV	19 (5.4%)	19 (5.4%)
unkown	8 (2.1%)	15 (4%)
Age, n (%)		
≤65	85 (22.9%)	79 (21.3%)
>65	99 (26.7%)	108 (29.1%)
unkown	3 (0.8%)	1 (0.3%)
Gender, n (%)		
Female	63 (16.8%)	71 (18.9%)
Male	124 (33.1%)	117 (31.2%)
unkown	0	0
Grade, n (%)		
G1	6 (1.6%)	4 (1.1%)
G2	81 (22.1%)	56 (15.3%)
G3	98 (26.8%)	121 (33.1%)
unkown	2 (0.5%)	7 (1.9%)

### Collection of Clinical Samples

Samples were collected from patients who underwent gastric cancer surgery in the Department of Gastroenterology, The First Affiliated Hospital of Bengbu Medical College, from January 2017 to December 2018. A total of eight gastric cancer tissue samples and their paraneoplastic tissues were collected for Western blot experiments and 197 gastric cancer tissue samples and 30 normal paraneoplastic tissues for immunohistochemical staining. No patients had received chemotherapy, radiotherapy or biological treatment before surgery and were diagnosed with gastric cancer before and after surgery. The collected tissue samples were stored in a −80°C refrigerator immediately after surgery until protein extraction.

### Experimental Antibodies

Rabbit anti-human antibody CHSY3 (100 μg) was obtained from OriGene China. The primary antibody to β-actin and CD3+/CD4+/CD8+ T cells alpha rabbit monoclonal antibody were provided by Cell Signaling Technology, Inc. (Danvers, MA, United States). Horseradish peroxidase (HRP)-conjugated anti-rabbit antibody was provided by Jackson ImmunoResearch Inc. (West Grove, PA, United States). Bovine serum albumin was obtained from Sigma-Aldrich (St. Louis, MO, United States). Skimmed milk and Tween-20 were purchased from Sangon Biotech Co., Ltd. (Shanghai, China).

### Immunohistochemical Staining

All samples were fixed in 4% paraformaldehyde, embedded in paraffin, sectioned to 4 μm and adhered to slides. After de-affinity under different density gradients of xylene, the slides were rehydrated, and antigens were retrieved with citric acid buffer (pH 7.8, 0.1 M) at approximately 82°C for 24 min. The sections were evenly covered with endogenous peroxidase blocking solution for 15 min at room temperature to block the activity of endogenous peroxidase. After incubation with gene primary antibody or overnight at 4°C, the slides were washed gently with phosphate-buffered saline and incubated with biotin-conjugated secondary antibody for 10 min at room temperature and incubated with streptavidin peroxidase for 5 min. All sections were stained with hematoxylin and then cleaned. After sections were dried and cleared, immunohistochemical evaluation was performed.

### Western Blot

Fresh gastric cancer tissue was obtained, and total protein was extracted. PowerPac HV high-voltage power supply (Bio-Rad Laboratories, Inc., CA, United States) was used for protein electrophoresis. Total protein was electrophoretically transferred to polyvinylidene fluoride membrane after sodium dodecyl sulphate polyacrylamide gel electrophoresis. After closure with fresh 5% skimmed milk, the membranes were incubated overnight at 4°C with CHSY3 primary antibody diluted at 1:800 and β-actin diluted at 1:3000. After washing with TBST, the membranes were incubated with horseradish peroxidase-coupled anti-rabbit immunoglobulin G diluted at 1:3000 for 2 h at room temperature. Finally, specific protein bands were detected for visualisation using the Bio-Rad Chemical XRS Imaging System (Bio-Rad Laboratories).

To quantify the differential expression of *CHSY3* in STAD and normal tissues, we extracted parameters from the results of Western blot experiments by ImageJ (version: 1.8.0) software and visualized them by GraphPad Prism (version: 9) software for bar chart.

### Prognostic Analysis of *CHSY3* in Stomach Adenocarcinoma

Correlation between *CHSY3* expression (Affymetrix ID: 242100_at) and overall survival (OS; *n* = 881), first progression (FP) (*n* = 645) and post-progression survival (PPS) (*n* = 503) were analysed in the Kaplan–Meier Plotter website (https://kmplot.com/analysis/) by selecting the ‘Gastric Cancer’ module. Database samples were divided into high and low expression groups based on the mean value of *CHSY3* expression (OS: cut-off value = 124, high = 234, low = 397; FP: cut-off value = 100, high = 258, low = 264; PPS: cut-off value = 125, high = 138, low = 246). The log-rank test was used to compare the survival differences between the two groups. We also used TCGA database to combine *CHSY3* with clinical factors (gender, age, grade, T, N, M and stage) in the univariate and multivariate Cox analyses to look for independent indicators of STAD prognosis. Finally, we used the “rms” R package to construct a nomogram survival prediction system to integrate *CHSY3* expression with clinical factors in each patient with STAD to predict 1-, 3- and 5-year survival rates. This system uses scoring criteria based on the magnitude of the regression coefficients of all independent variables and gives each independent variable a score for each value considered. A total score can be calculated for each patient. Subsequently, the probability of outcome occurrence for each patient was calculated using a conversion function between the scores and probability of outcome occurrence (Iasonos et al., 2008; Balachandran et al., 2015). In addition, the accuracy of the nomogram prediction system in predicting 1-, 3- and 5-year survival rates was assessed using calibration curves. If the model prediction curve matched, located above or found below the reference line, the predicted value was considered equal, greater or lower than the actual value, respectively.

### Methylation Analysis of *CHSY3* in Stomach Adenocarcinoma

*CHSY3* expression-associated methylation sites were analysed using MEXPRESS (https://mexpress.be) by selecting the “STAD” section (*p*-value < 0.05). Then, SurvivalMeth (http://bio-bigdata.hrbmu. edu.cn/survivalmeth/) was used to analyse survival differences between the *CHSY3* expression-related methylation site and STAD prognosis (log-rank test, *p*-value < 0.05).

### Gene Set Enrichment Analysis

To further explore the oncogenic correlation between *CHSY3* and STAD, we used GSEA for oncogenic pathway enrichment. GSEA ranks genes according to the degree of differential expression in the two types of samples using a predefined set of genes, usually from functional annotations or results of previous experiments, and then tests whether the predefined set of genes is enriched at the top or bottom of this ranking table (Subramanian et al., 2005). Gene sets under pathways with |normalised enrichment score |>1, nominal *p*-value < 0.05 and false discovery rate q-value < 0.25 are generally considered significant.

### Analysis of the Role of *CHSY3* in the TIME of Stomach Adenocarcinoma

The Hallmark consensus pathway gene set downloaded from the Molecular Signatures Database (http://www.gsea-msigdb.org/gsea/login.jsp) was subjected to gene set variation analysis (GSVA) using the “GSVA” R language package to assess the enrichment of the Hallmark pathway in the high and low *CHSY3* expression groups (*p*-value < 0.05). GSVA is a non-parametric, unsupervised algorithm that assesses the enrichment of different metabolic pathways between samples by converting the sample-to-sample gene expression matrix into a sample-to-sample genomic expression matrix (Hänzelmann et al., 2013). Single-sample GSEA (ssGSEA) enrichment analysis was also performed using the “GSVA” R language package to assess the differences in immune cells and functions between the high and low *CHSY3* expression groups (*p*-value < 0.05).

The estimation of stromal and immune cells in malignant tumour tissues using expression data (ESTIMATE) algorithm was used to assess the immune cell score, stromal score and tumour purity score of the high and low *CHSY3* expression groups in the TIME of STAD. ESTIMATE analysis was performed using transcriptional profiles of cancer samples to evaluate the number of tumour cells, infiltrating immune cells and stromal cells, and the “estimateScore” function was used to calculate tumour purity, immune cell score and stromal cell score of all samples (*p*-value < 0.05).

### Construction of *CHSY3*-Related Immune Signatures

The “limma” R language package was used to analyse *CHSY3*-related marker genes in high and low *CHSY3* expression groups (*p*-value < 0.05). Sixty-six immune cell surface marker genes in STAD were analysed by the “reshape2” R package to determine correlation with *CHSY3* expression (*p*-value < 0.05). The prognosis of *CHSY3*-related marker genes in STAD was further analysed using prognosis-related genes for the stepwise multivariate Cox proportional hazard regression analysis to obtain the optimal candidates and construct an immune-related risk model. The formula for calculating the risk score was as follows:

$$Risks\;core=\sum_{i=1}^{n}coefi\times Xi$$

The “coefi” and “Xi” represent the coefficient and expression level of each *CHSY3* prognosis-related marker gene, respectively. According to the risk score of the model, TCGA samples can be divided into the high- and low-risk groups. The log-rank test was used to compare the survival differences between the two groups (*p*-value < 0.05). The “survivalROC” package was used to performreceiver operating characteristic (ROC) curve, and the Area Under Curve (AUC) values were obtained to evaluate the prognostic model’s reliability. To further analyse the prognostic risk of model scores, we performed univariate and multivariate Cox analyses by combining model scores with clinical factors (sex, age, grade, T, N, M, and stages) to assess whether model scores could be used as independent prognostic factors. Finally, the Tumor Immune Dysfunction and Exclusion (TIDE) (http://tide.dfci.harvard.edu/login/) was used to evaluate the risk of immune evasion and the effect of immunotherapy between the high- and low-risk groups of the model.

### Statistical Analysis

Statistical analyses were performed using the R software version 4.1.1 (R Foundation for Statistical Computing, Vienna, Austria). For quantitative data in the article data analysis, the significance of normally distributed variables was estimated using Student’s t-test, and non-normally distributed variables were analysed using the Wilcoxon rank-sum test. The log-rank test was used to compare data between two groups, and the Kruskal–Wallis test was performed to compare data between more than two groups. A *p*-value < 0.05 was considered significant.

## Results

### Expression Analysis of *CHSY3* in Stomach Adenocarcinoma

**FIGURE 1:**
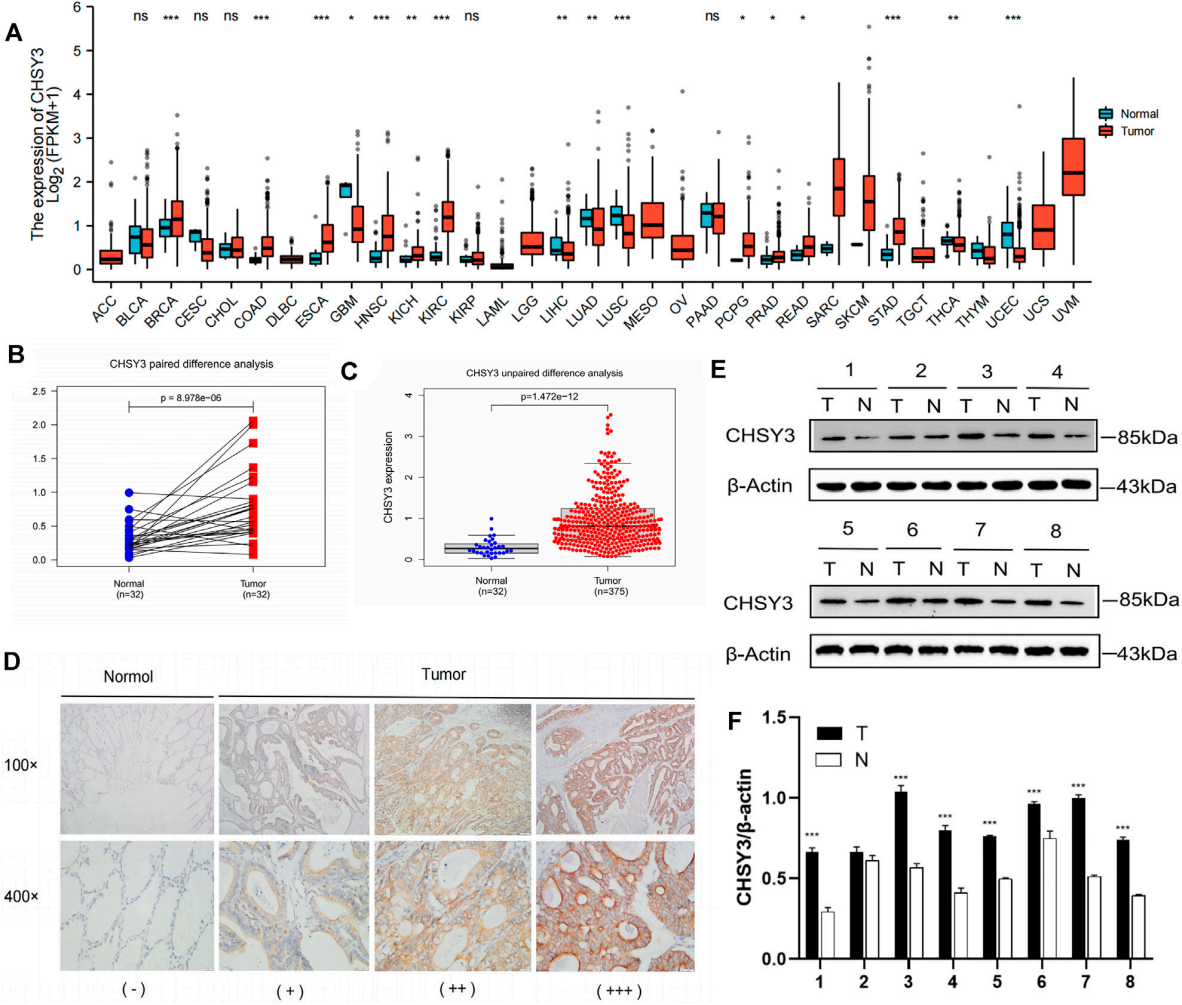
CHSY3 expression levels in tumours. **(A)** Differntial expression of CHSY3 in tumour tissues and normal tissues in pan-cancer. CHSY3 expression was significantly higher in STAD than in normal tissue in TCGA database (p-value<0.05). **(B)** Paired and **(C)** unpaired differentail analyses. **(D)** Immunohistochemical staining analysis showing higher expression in STAD than in normal adjacent tissue. **(E)** Western blot experiment comparing CHSY3 expression in tumour and normal tissues. **(F)** Quantitative difference analysis between tumour tissue and normal tissue for western blot experiment results. *p-value<0.05; **p-value<0.01; ***p-value<0.001.

Pan-cancer differential expression analysis revealed that *CHSY3* was significantly differentially expressed in various cancers, with significant down-regulation in glioblastoma multiforme, lung adenocarcinoma, lung squamous carcinoma, etc., and significant up-regulation in STAD, breast-invasive carcinoma, kidney renal clear cell carcinoma, etc. (*p*-value < 0.05, Figure 1A). Paired difference analysis demonstrated significantly higher *CHSY3* expression in STAD tissues than in paracancerous tissues (*p*-value < 0.05, Figure 1B) and exhibited consistent results in the unpaired difference analysis (*p*-value < 0.05, Figure 1C). At the tissue level, *CHSY3* expression was higher in STAD tissues than in paraneoplastic tissues by immunohistochemical staining analysis (Figure 1D). This result was also observed at the protein level by Western blot experiments (Figure 1E). In addition, quantitative analysis of Western blot experiments demonstrated that *CHSY3* expression was significantly higher in tumour tissues than in normal tissues in 7 out of 8 pairs of tissues (Figure 1F). Altogether, *CHSY3* expression was significantly higher in STAD than in normal tissues.

### Prognostic Analysis and Assessment of the Practical Clinical Utility of *CHSY3* in Stomach Adenocarcinoma

**FIGURE 2:**
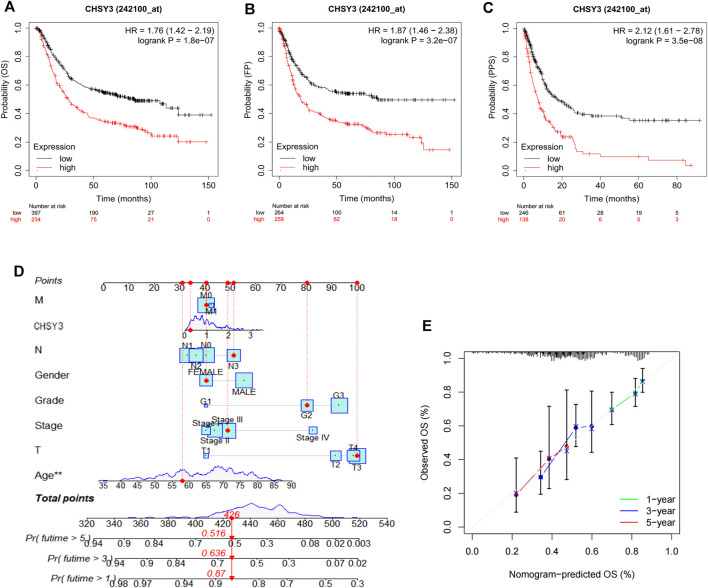
Prognostic analysis of CHSY3 in STAD in the Kaplan-Meier Plotter database. In STAD, the survival of the low CHSY3 expression group was significantly better than that of the high CHSY3 expression group (p-value<0.05). A low CHSY3 expression was associated with better overall survival **(A)**, first progession **(B)** and post-progession survival **(C)**. **(D)** Integration of CHSY3 expression and clinical factors to construct nomogram survival predictions. Each factor in the prediction system corresponds to a score, and th sum of the scores for all clinical factors corresponds to the total patient score, thus predicting 1-3, and 5-year survival rates. **(E)** Calibration curves for 1-3- and 5 year survival predictions in the nomogram prediction system.

The survival analysis revealed that the high *CHSY3* expression group had poorer OS (Figure 2A), FP (Figure 2B) and PPS (Figure 2C) than the low *CHSY3* expression group in the Kaplan–Meier Plotter database (*p*-value < 0.05). In addition, the univariate Cox analysis presented that T3, T4, N1, N3, M1, stage III, stage IV, age >65 years and *CHSY3* expression were all risk factors significantly associated with prognosis (*p*-value < 0.05). The multivariate Cox analysis indicated that age and *CHSY3* could be used as risk factors for STAD independent of the clinical factors in Table 2 (*p*-value < 0.05). This implies that *CHSY3* can be used as an indicator of STAD prognosis.

**TABLE 2:** Univariate and multivariate COX analysis of CHSY3 and clinical factors in the TCGA database.

Characteristics	Total(N)	Univariate analysis		Multivariate analysis	
		HR (95% CI)	*p*-value	HR (95% CI)	*p*-value
T stage	362				
T1	18	References			
T2	78	6.725 (0.913–49.524)	0.061	4.079 (0.515–32.293)	0.183
T3	167	9.548 (1.326–68.748)	**0.025**	4.928 (0.560–43.356)	0.151
T4	99	9.634 (1.323–70.151)	**0.025**	4.121 (0.453–37.528)	0.209
N stage	352				
N0	107	References			
N1	97	1.629 (1.001–2.649)	**0.049**	1.262 (0.623–2.554)	0.519
N2	74	1.655 (0.979–2.797)	0.060	1.422 (0.598–3.383)	0.426
N3	74	2.709 (1.669–4.396)	**<0.001**	1.987 (0.835–4.726)	0.120
M stage	352				
M0	327	References			
M1	25	2.254 (1.295–3.924)	**0.004**	1.194 (0.505–2.824)	0.686
Stage	347				
Stage I	50	References			
Stage II	110	1.551 (0.782–3.078)	0.209	1.047 (0.370–2.964)	0.931
Stage III	149	2.381 (1.256–4.515)	**0.008**	1.084 (0.272–4.320)	0.909
Stage IV	38	3.991 (1.944–8.192)	**<0.001**	2.328 (0.566–9.575)	0.241
Gender	370				
Female	133	References			
Male	237	1.267 (0.891–1.804)	0.188		
Age	367				
≤65	163	References			
>65	204	1.620 (1.154–2.276)	**0.005**	1.769 (1.219–2.568)	**0.003**
Grade	361				
G1	10	References			
G2	134	1.648 (0.400–6.787)	0.489		
G3	217	2.174 (0.535–8.832)	0.278		
CHSY3	370				
Low	184	References			
High	186	1.508 (1.082–2.103)	**0.015**	1.473 (1.025–2.115)	**0.036**

Bold: *p*-value < 0.05.

To further evaluate the practical benefits of *CHSY3* in clinical applications, we integrated *CHSY3* expression data in STAD with clinical factors to construct a nomogram to predict patient survival at 1, 3 and 5 years (Figure 2D). The nomogram system survival prediction curves have a high coincidence with the calibration curve, which indicates the high accuracy of our nomogram (Figure 2E). Therefore, the proposed nomogram survival prediction system has a good clinical utility value.

### Analysis of *CHSY3*-Related Methylation Sites and Their Prognosis

**FIGURE 3:**
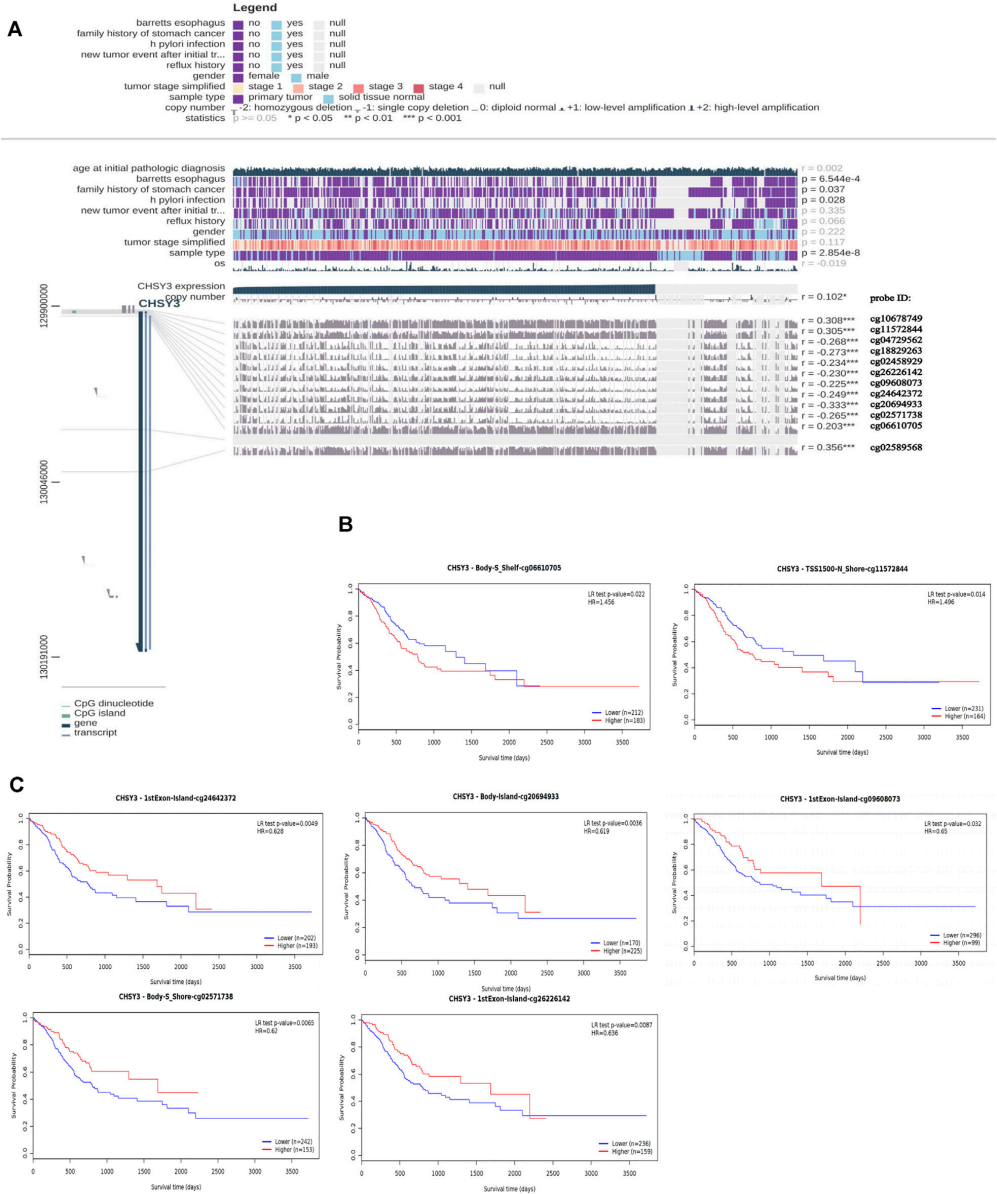
Correlation and prognostic analysis of CHSY3 methylation sites in STAD. **(A)** Methylation sites associated with CHSY3 expression inSTAD. r: correlation; probe ID: methylation site; *p-value<0.05; **p-value<0.01; ***p-value<0.001. **(B)** The survival of the high expression group was significantly poorer than that of the low expression group at the CHSY3 positively correlated methylation sites. **(C)** The survival of the low expression group was significantly poorer than that of the low expression group at the CHSY3 negatively associated methylation sites.

MEXPRESS identified a total of 12 methylation sites associated with *CHSY3* expression in STAD. Among these *CHSY3* expression-associated methylation sites, cg10678749, cg11572844, cg02589568, and cg06610705 presented a significant positive correlation with *CHSY3* expression (r > 0, *p*-value < 0.05), while cg04729562, cg18829263, cg02458929, cg26226142, cg09608073, cg24642372, cg20694933, and cg02571738 (r < 0, *p*-value < 0.05) had a significant negative correlation with *CHSY3* expression (Figure 3A). SurvivalMeth revealed that seven *CHSY3* expression-associated methylation sites had significant survival value. Cg06610705 and cg11572844 were positively correlated with *CHSY3* expression, and high *CHSY3* expression groups of these sites had a poorer prognosis than the low *CHSY3* expression groups (Figure 3B, *p*-value < 0.05). Cg24642372, cg20694933, cg09608073, cg02571738 and cg26226142 were negatively correlated with *CHSY3* expression, of which the low *CHSY3* expression groups had a poorer prognosis than the high *CHSY3* expression groups (Figure 3C, *p*-value < 0.05). Consistent with previous findings, the group with high *CHSY3* expression-associated methylation sites had a poor prognosis of STAD.

### GSEA Oncogenic Pathway Enrichment

**FIGURE 4:**
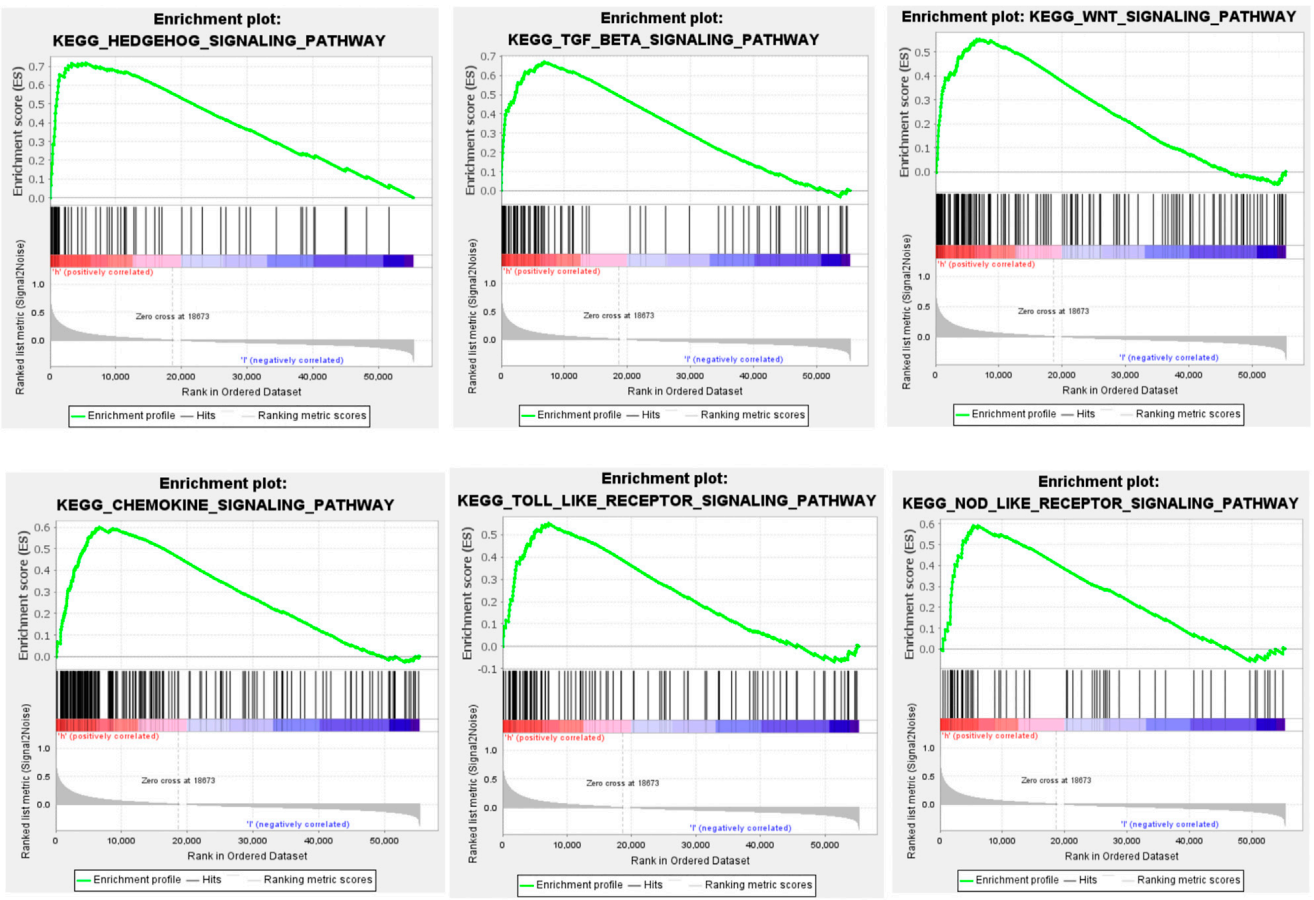
GSEA of the oncogenic pathway up-regulated by CHSY3 in STAD.

GSEA showed that *CHSY3* is involved in multiple signalling pathways in STAD. Table 3 exhibits the top 10 up-regulated signalling pathways associated with *CHSY3* according to the GSEA score in STAD. We found that the transforming growth factor-beta (TGF-β), Wnt and Hedgehog signalling pathways were associated with the tumour mesenchymal pathway. The chemokine, Toll-like receptor and NOD-like receptor signalling pathways are involved in immune and inflammatory activation (Figure 4). Interestingly, high expression of *CHSY3* in STAD did not perform a better prognosis.

**TABLE 3:** CHSY3 oncogenic pathway parameters in GSEA enrichment analysis.

GeneSet	NES	NOM *p*-val	FDR q-val
HEDGEHOG_SIGNALLING_PATHWAY	2.31	0	0
TGF_BETA_SIGNALLING_PATHWAY	2.26	0	0
MAPK_SIGNALLING_PATHWAY	2.25	0	0
CHEMOKINE_SIGNALLING_PATHWAY	2.12	0	0.001
JAK_STAT_SIGNALLING_PATHWAY	2.10	0	0.001
WNT_SIGNALLING_PATHWAY	2.00	0.006	0.004
NEUROTROPHIN_SIGNALING_PATHWAY	1.91	0	0.012
ADIPOCYTOKINE_SIGNALING_PATHWAY	1.90	0	0.012
TOLL_LIKE_RECEPTOR_SIGNALING_PATHWAY	1.90	0.006	0.012
NOD_LIKE_RECEPTOR_SIGNALING_PATHWAY	1.89	0.006	0.011

### Effect of *CHSY3* Expression on the TIME of Stomach Adenocarcinoma

**FIGURE 5:**
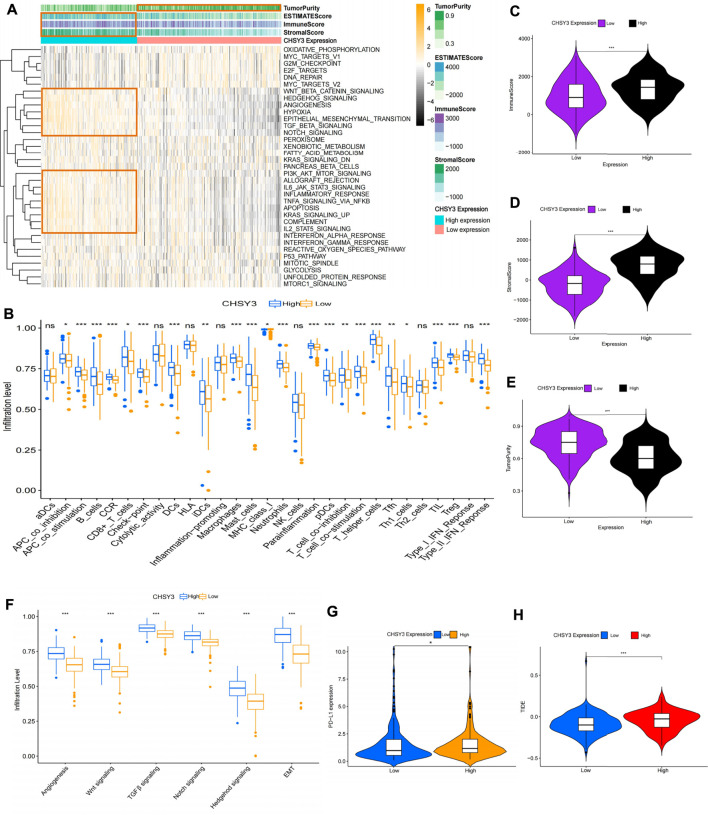
Analysis of the immune microenvironment of CHSY3 in STAD. **(A)** Enrichment of high and low CHSY3 expression groups in STAD in the Hallmark pathway gene sets. Yellow black represent samples with high and low expressions, respectively. **(B)** Differential analysis of high and low CHSY3 expression groups in STAD regrading immune cells and functions. Red and blue represent samples with high and low expressions, respectively. The ESTIMATE algorithm assesses the immune cell score **(C)**, stromal score **(D)** and tumour purity **(E)** score in the high and low CHSY3 expression groups in STAD. **(F)** DIfferential analysis of high and low CHSY3 expression groups in STAD stromal signalling pathway. **(G)**, **(H)** Assessment of immune evasion and immunotherapy efficacy in high and low CHSY3 expression groups. *p-value<0.05; **p-value<0.01; ***p-value<0.001.

To explore the effect of *CHSY3* expression on the TIME in STAD, the Hallmark pathway gene set was enriched in the high and low *CHSY3* expression groups using GSVA enrichment analysis. The results presented that the high *CHSY3* expression group was up-regulated in immune activation and inflammatory signalling pathways, such as the TNFA signalling via NF-KB, allograft rejection, complement, IL6–JAK–STAT3 signalling, IL2–STAT5 signalling and inflammatory response. Moreover, the high *CHSY3* expression group was up-regulated in epithelial–mesenchymal transition (EMT), TGF-β signalling, angiogenesis, Wnt/beta-catenin signalling, Notch signalling and other mesenchymal signalling pathways, while the low *CHSY3* expression group demonstrated the opposite phenomenon (Figure 5A). The heatmap and differential analysis of immune cells and functions in STAD using the ssGSEA method demonstrated higher enrichment in the high *CHSY3* expression group than in the low *CHSY3* expression group (Figures 5A,B). The ESTIMATE assessment of the TIME in STAD also revealed that the immune cell score (Figures 5A,C) and tumour stroma score (Figures 5A,D) were significantly higher in the high *CHSY3* expression group than in the low *CHSY3* expression group, while the tumour purity score (Figures 5A,E) demonstrated the opposite phenomenon. These analyses consistently revealed that the high *CHSY3* expression group was associated with severe immune cell infiltration. However, this immune advantage did not exhibit a survival advantage, which became the focus of our attention. Studies have demonstrated that the immune-exclusive tumour phenotype is characterised by numerous immune cells that are retained in the stroma surrounding the nest of tumour cells without penetrating these cells (Chen and Mellman, 2017). Immune-evading tumours are generally characterised by high TGF-β expression, myeloid inflammation and tumour neovascularisation as microenvironmental features (Fukumura et al., 2018; Metelli et al., 2018; Hegde and Chen, 2020). Figure 5F displays that signalling pathways such as EMT, angiogenesis, TGF-β and Wnt were significantly higher in the high *CHSY3* expression group than in the low *CHSY3* expression group. Therefore, a high *CHSY3* expression mediates TIME immune evasion in STAD.

Currently, programmed death-ligand 1 (*PD-L1*) is a remarkable discovery in immunotherapy, and its expression level is an important predictor of the response to anti-PD-1/L1 therapy (Fukumura et al., 2018). We further selected *PD-L1* to assess the effect of immunotherapy, and the results revealed that *PD-L1* expression was higher in the high *CHSY3* expression group than in the low *CHSY3* expression group, implying that the high *CHSY3* expression group may have immune-evasion ability and better immunotherapy effects (Figure 5G). Studies have also reported that the newly discovered TIDE score is an effective predictor of anti-*PD1* and anti-*CTLA4* therapy among all efficacy characteristics of ICI suppression therapy (Jiang et al., 2018). The predictive function of the TIDE score for efficacy was stable regardless of the degree of tumour-infiltrating cytotoxic T-cells (Jiang et al., 2018). The TIDE score analysis indicated that the high *CHSY3* expression group had significantly higher score than the low *CHSY3* expression group; this finding also demonstrated that high *CHSY3* expression mediates the immune-evasion status of the TIME in STAD (Figure 5H).

### Immunoprognostic Analysis of *CHSY3* in Stomach Adenocarcinoma

**FIGURE 6:**
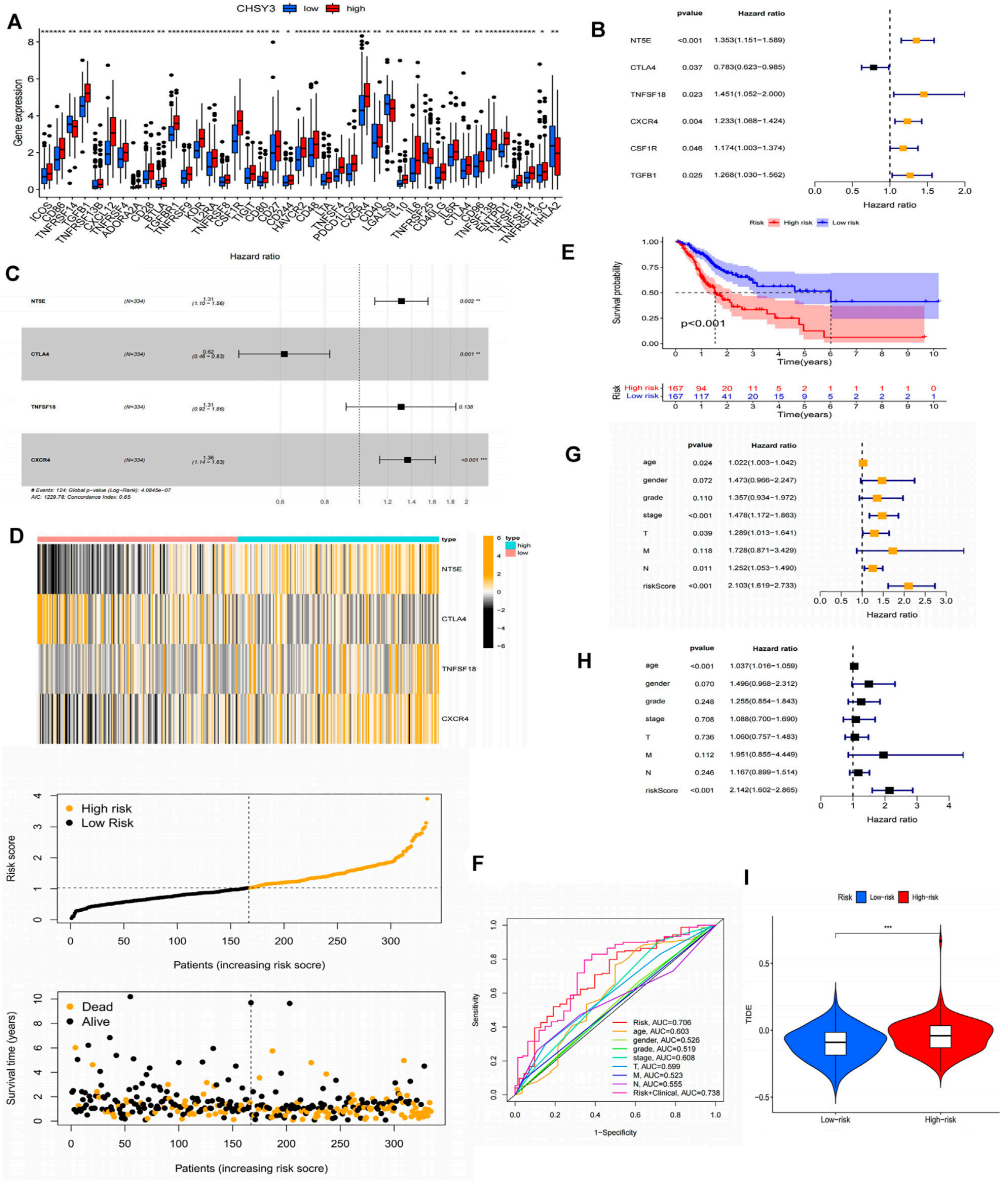
Construction of a CHSY3 immunomodulator risk model in STAD. **(A)** Diffenential analysis of high and low CHSY3 expression groups in immunomodulator. **(B)** prognostic immunomodulators associated with CHSY3 in STAD. **(C)**, **(D)** Construction of a cox risk proportional regression model for CHSY3 associated with immunomodulators in STAD. **(E)** The prognosis for the high-risk group divided according to the risk model is significantly poorer than that for the low risk group. **(F)** Assesing the accuracy of Cox model risk predictions using ROC curves. Model risk scores combined with clinical factors for **(G)** univariate and **(H)** multivariate COX analysis. **(I)** assesment of immune evasion and immunotherapy efficacy in high-risk and low-risk groups. *p-value<0.05; **p-value<0.01; ***p-value<0.001.

Using the PubMed database, we retrieved 66 marker genes localised on the surface of immune cells. Proteins encoded by these genes, also known as immunomodulators, are classified as immunostimulators and immunoinhibitors, and studies have demonstrated that immunomodulators have significant effects on prognosis (Hadden, 1993; Hengge et al., 2001). In the correlation analysis, 46 of 66 immunomodulators in STAD were associated with *CHSY3* expression (Table 4). In the differential expression analysis, 41 of 46 *CHSY3*-related immunomodulators were significantly different between the high and low *CHSY3* expression groups (Figure 6A, *p*-value < 0.05). In the Cox survival analyses, *CHSY3* expression-related genes, namely, *CSF1R*, *TGFB1*, *CXCR4*, and *TNFSF18*, were prognosis-related risk factors, while CTLA4 was a prognosis-related favourable factor (Figure 6B, *p*-value < 0.05). Moreover, we constructed a *CSHY3*-related immune risk model based on prognostic immunomodulators. The model risk scoring formula is as follows: risk core = (coefficient × *CXCR4* expression) + (coefficient × *CTLA4* expression) (Figure 6C). The immune risk model was constructed to classify TCGA samples according to risk and prognosis (Figure 6D). According to the model, the survival of the high-risk group was significantly worse than that of the low-risk group (Figure 6E). The accuracy assessment of the ROC curve for the risk model revealed that the risk scoring of the model (AUC = 0.706) and the model risk scoring combined with clinical factors (AUC = 0.738) had a high accuracy. In addition, the univariate Cox analysis bared that age, stage, T, N and risk score were prognostically relevant risk factors (Figure 6G), and the multivariate Cox analysis revealed that age and risk score could be used as independent prognostic risk factors (Figure 6H). These analyses suggest that the *CHSY3*-mediated TIME of STAD is associated with a poor prognosis. The TIDE score indicated that the high-risk group demonstrated a significantly higher risk of immune evasion than the low-risk group (Figure 6I). This is consistent with the previous conclusion that *CHSY3* mediates the immune-evasion status in the TIME of STAD.

**TABLE 4:** Immune checkpoints associated with CHSY3 in STAD.

Gene	Cor	*p*-value	Gene	Cor	*p*-value
ADORA2A	0.2799	**0**	ICOS	0.2094	**0**
BTLA	0.1579	**0.0022**	ICOSLG	−0.0218	0.6743
CD160	0.0155	0.7649	IL2RA	0.2785	**0**
CD244	0.1345	**0.0092**	IL6	0.4157	**0**
CD274	0.0965	0.0619	IL6R	0.1941	**0.0002**
CD96	0.1613	**0.0017**	KLRC1	0.0931	0.0719
CSF1R	0.4609	**0**	KLRK1	0.0859	0.0966
CTLA4	0.1795	**0.0005**	LTA	0.1666	**0.0012**
HAVCR2	0.3679	**0**	MICB	0.0329	0.5251
IL10	0.4177	**0**	NT5E	0.1331	**0.0099**
IL10RB	−0.0516	0.3184	PVR	0.0323	0.5332
KDR	0.5242	**0**	RAET1E	−0.0118	0.8198
LAG3	0.0736	0.1549	TMIGD2	0.0044	0.9325
LGALS9	−0.2291	**0**	TNFRSF13B	0.1487	**0.0039**
PDCD1	0.1164	**0.0242**	TNFRSF13C	0.1059	**0.0405**
PDCD1LG2	0.4484	**0**	TNFRSF14	−0.1577	**0.0022**
TGFB1	0.5199	**0**	TNFRSF17	0.0525	0.3101
TGFBR1	0.5119	**0**	TNFRSF18	0.0797	0.1232
TIGIT	0.1645	**0.0014**	TNFRSF25	−0.1139	**0.0274**
VTCN1	−0.0278	0.5912	TNFRSF4	0.3171	**0**
CD27	0.1372	**0.0078**	TNFRSF8	0.274	**0**
CD28	0.3438	**0**	TNFRSF9	0.2344	**0**
CD40	0.2148	**0**	TNFSF13	−0.084	0.1042
CD40LG	0.1662	**0.0013**	TNFSF13B	0.2206	**0**
CD48	0.2187	**0**	TNFSF14	0.245	**0**
CD70	0.1336	**0.0096**	TNFSF15	0.0429	0.4069
CD80	0.3079	**0**	TNFSF18	0.2101	**0**
CD86	0.3678	**0**	TNFSF4	0.4995	**0**
CXCL12	0.5547	**0**	TNFSF9	0.0869	**0.0931**
CXCR4	0.3678	**0**	ULBP1	0.0618	0.2323
ENTPD1	0.6211	**0**	KIR2DL1	0.105	**0.0421**
HHLA2	−0.1329	**0.01**	KIR2DL3	0.1581	**0.0021**

Bold: *p*-value < 0.05.

### Immune Cell Infiltration in *CHSY3* High and Low Expression Groups

**FIGURE 7:**
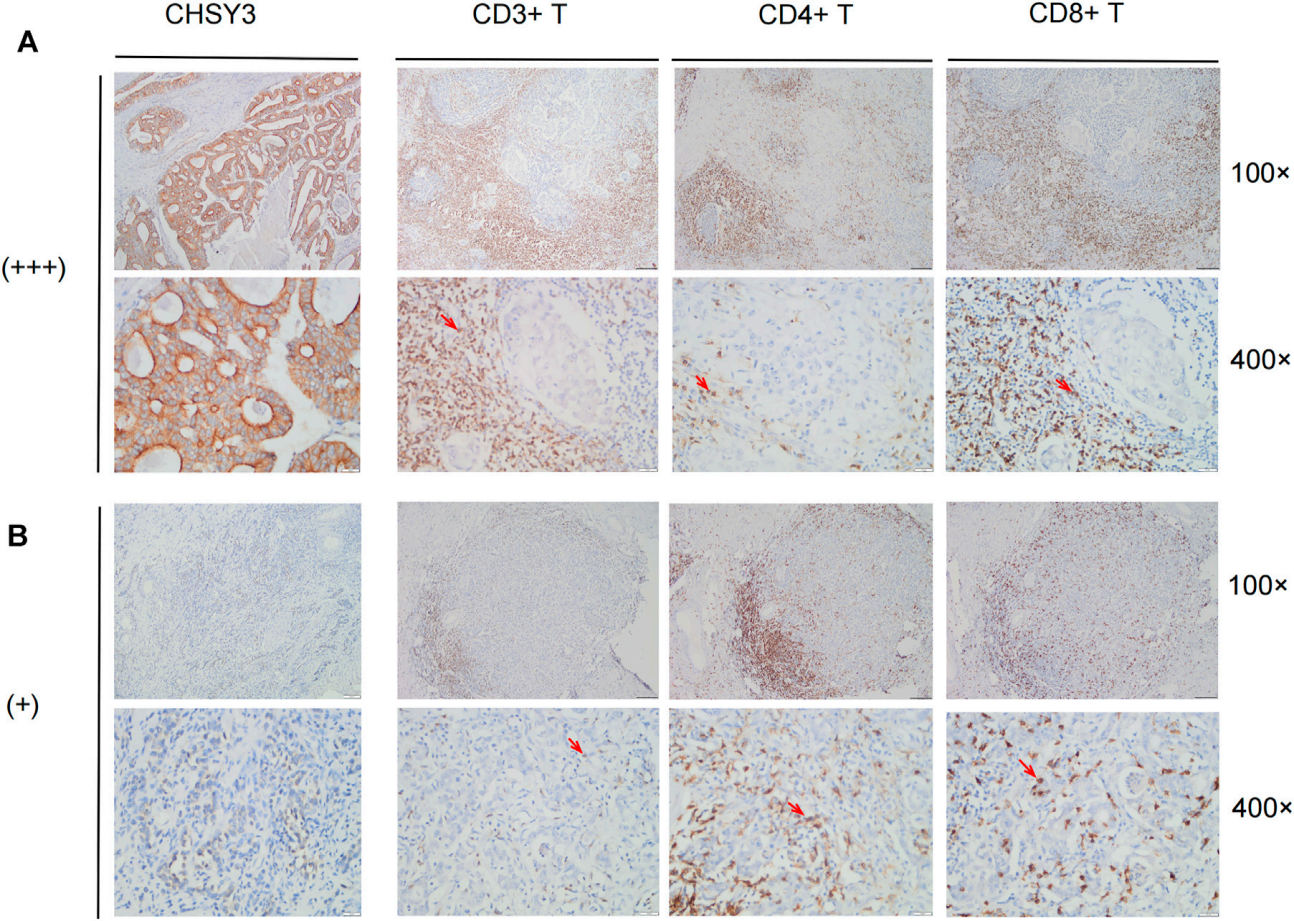
Immune cell infiltration in CHSY3 high and low expression groups. **(A)** Infiltation CD3+ T cells, CD4+ T cells and CD8+ T cells in CHSY3 high expression group (+++). **(B)** Infiltration of CD3+ T cells, CD4+ T cells and CD8+ T cells in CHSY3 low expression goup (+). Red arrow: immune cells location.

To further verify that *CHSY3* mediates TIME immune evasion in STAD, we selected tissue samples expressing “+” and “+++” of *CHSY3* in STAD in Figure 1D for immunohistochemical staining analysis. The purpose of staining was to observe the infiltration of CD3^+^ T cells, CD4^+^ T cells and CD8^+^ T cells in STAD tumour tissues and surrounding tissues to determine whether the high expression of *CHSY3* in TIME is consistent with the characteristics of immune evasion. The results showed that CD3^+^ T cells, CD4^+^ T cells and CD8^+^ T cells in *CHSY3* expressing “+++” tissues were mainly clustered in the tumour peripheral stroma, with few immune cells penetrating the stroma into the tumor parenchyma (Figure 7A). In contrast, in *CHSY3* expressing “+”, tumour peripheral CD3^+^ T cells, CD4^+^ T cells and CD8^+^ T cells clustered less and more immune cells penetrated the stroma into the tumour parenchyma (Figure 7B). In summary, this phenomenon further confirms that the *CHSY3* high expression group mediates immune evasion in the TIME of STAD.

## Discussion

Glycosylation is an important feature of tumours, and the occurrence and development of malignant tumours are often associated with abnormal glycosylation (Stowell et al., 2015). The chondroitin sulphate synthase family, an important enzyme-regulating glycosylation, is closely involved in cancer development (Pinho and Reis, 2015). *CHSY1* overexpression in hepatocellular carcinoma promotes cancer cell growth, migration, invasion and EMT through the hedgehog signalling pathway (Liu et al., 2017). Tumorigenesis and choriocarcinoma metastasis were significantly inhibited by *CHSY2* knockdown in choriocarcinoma (Zhang et al., 2021). However, only a few studies have focused on *CHSY3* in cancer. To the best of our knowledge, we demonstrated for the first time that *CHSY3* is overexpressed in STAD through TCGA database and immunohistochemical staining and Western blot experiments. The survival analysis indicated that a high *CHSY3* expression was associated with a poor prognosis, and the multivariate Cox analysis indicated that *CHSY3* could be an independent prognostic risk factor for STAD. The survival analysis of *CHSY3*-associated methylation sites in STAD also consistently demonstrated a poor prognosis. These findings suggest that *CHSY3* can be used as a biomarker of poor prognosis in STAD.

Studies have demonstrated that altered glycosylation can modulate the inflammatory response, promote cancer cell metastasis, regulate apoptosis and contribute to tumour immune escape (RodrÍguez et al., 2018; Läubli and Borsig, 2019; Reily et al., 2019). Compared with normal cells, tumour cells have a different “glycosylation coating” (RodrÍguez et al., 2018). Since immune cells express different glycosylation-dependent lectin receptors, they can sense changes in glycosylation markers in their environment and respond accordingly, which may lead to immunosuppression (RodrÍguez et al., 2018). In this study, *CHSY3* was found to up-regulate immune activation and mesenchymal signalling pathways in STAD, and our findings were further supported by analysis of the Hallmark pathway gene set and TIME evaluation using the ESTIMATE algorithm. The immune-evasion phenotype is generally characterised by the activation of mesenchymal signalling pathways, such as high TGF-β expression, myeloid inflammation and tumour neovascularisation (Fukumura et al., 2018; Metelli et al., 2018; Hegde and Chen, 2020). A high TGF-β expression suppresses the activity of CD8^+^ T-cells, dendritic cells, natural killer cells and other key immune cells in anti-tumour immunity in the TIME, while promoting the action of regulatory T-cells, thus making the entire TIME suppressive (Batlle and Massagué, 2019; Liu et al., 2020; Derynck et al., 2021). Therefore, given the high *CHSY3* expression, the activation of the tumour mesenchyme inhibit numerous T-cells to infiltrate the tumour through the mesenchyme, resulting in an immune-evasion state. This is consistent with the poor prognostic results of our analysis. In addition, *PD-L1* and TIDE immune assessment demonstrated that a high *CHSY3* expression was associated with a significantly higher risk of immune evasion than low *CHSY3* expression. Finally, we performed immunohistochemical staining of CD3^+^ T cells, CD4^+^ T cells, and CD8^+^ T cells in STAD tissue samples. We demonstrated that the tumour stroma in the high *CHSY3* expression group had a large concentration of immune cells and few immune cells scattered in the parenchyma, while the tumour stroma in the low *CHSY3* expression group had less concentration of immune cells and more infiltration of immune cells in the parenchyma. Overall, our study demonstrates that high *CHSY3* expression mediates the immune-evasion status of the TIME in STAD.

ICs are divided into two main categories, namely, inhibitors and activators. ICI therapy represented by *PD-1* and *CTLA-4* inhibitors has undoubtedly caused a breakthrough in anti-tumour therapy (Topalian et al., 2016; Darvin et al., 2018; Kalbasi and Ribas, 2020). *PD-1/CTLA4* are immune cell surface marker proteins, and tumour cells cause immune evasion by altering the functions of these proteins (Pardoll, 2012; Sun et al., 2018). In this study, we analysed immune cell surface marker proteins associated with *CHSY3* expression and constructed Cox proportional regression models using prognosis-related marker genes. Accordingly, the survival of the high-risk group was significantly lower than that of the low-risk group, and the model risk score could be used as an independent prognostic risk score factor. TIDE scores also showed that the high-risk group had significantly higher immune-evasion ability than the low-risk group. This also demonstrated that *CHSY3* affects the TIME of STAD leading to a poor prognosis.

In conclusion, *CHSY3* can be used as a biomarker of poor STAD prognosis and mediates immune-evasion status in STAD.

## Data Availability

The original contributions presented in the study are included in the article/Supplementary Material, further inquiries can be directed to the corresponding authors.

## Abbreviations

EMT, epithelial–mesenchymal transition; ESTIMATE, Estimation of STromal and Immune cells in MAlignant Tumour tissues using Expression data; FP, first progression; GSEA, Gene Set Enrichment Analysis; GSVA, gene set variation analysis; OS, overall survival; PET-CT, positron emission computed tomography-computed tomography; PPS, post-progression survival; ssGSEA, single-sample gene set enrichment analysis; STAD, stomach adenocarcinoma; TCGA, The Cancer Genome Atlas; TGF-β, TGF-beta; TIDE, Tumor Immune Dysfunction and Exclusion; TIME, tumour immune microenvironment; AUC, Area Under Curve; ROC, receiver operating characteristic.
